# Supplementary material for: Resilience of BST-2/Tetherin structure to single amino acid substitutions
Source: PeerJ. 2019 May 31;7:e7043. doi: 10.7717/peerj.7043 (PMC6546079; doi:10.7717/peerj.7043)
Supplement: Figure S1 — Secondary structure over time plots from replicates of the WT, A100P and I120F simulations. Secondary structures were assigned by YASARA based on hydrogen bonding distances and psi angles at 25 picosecond intervals. ‘H’ stands for alpha helix, ‘T’ for turn, ‘C’ for coil, and ‘G’ for 3/10 helix. [file peerj-07-7043-s001.pdf]

Supplemental Figure 1

for Resilience of BST-2/Tetherin structure to single amino acid substitutions

by Ian R. Roy, Camden K. Sutton, and Christopher E. Berndsen

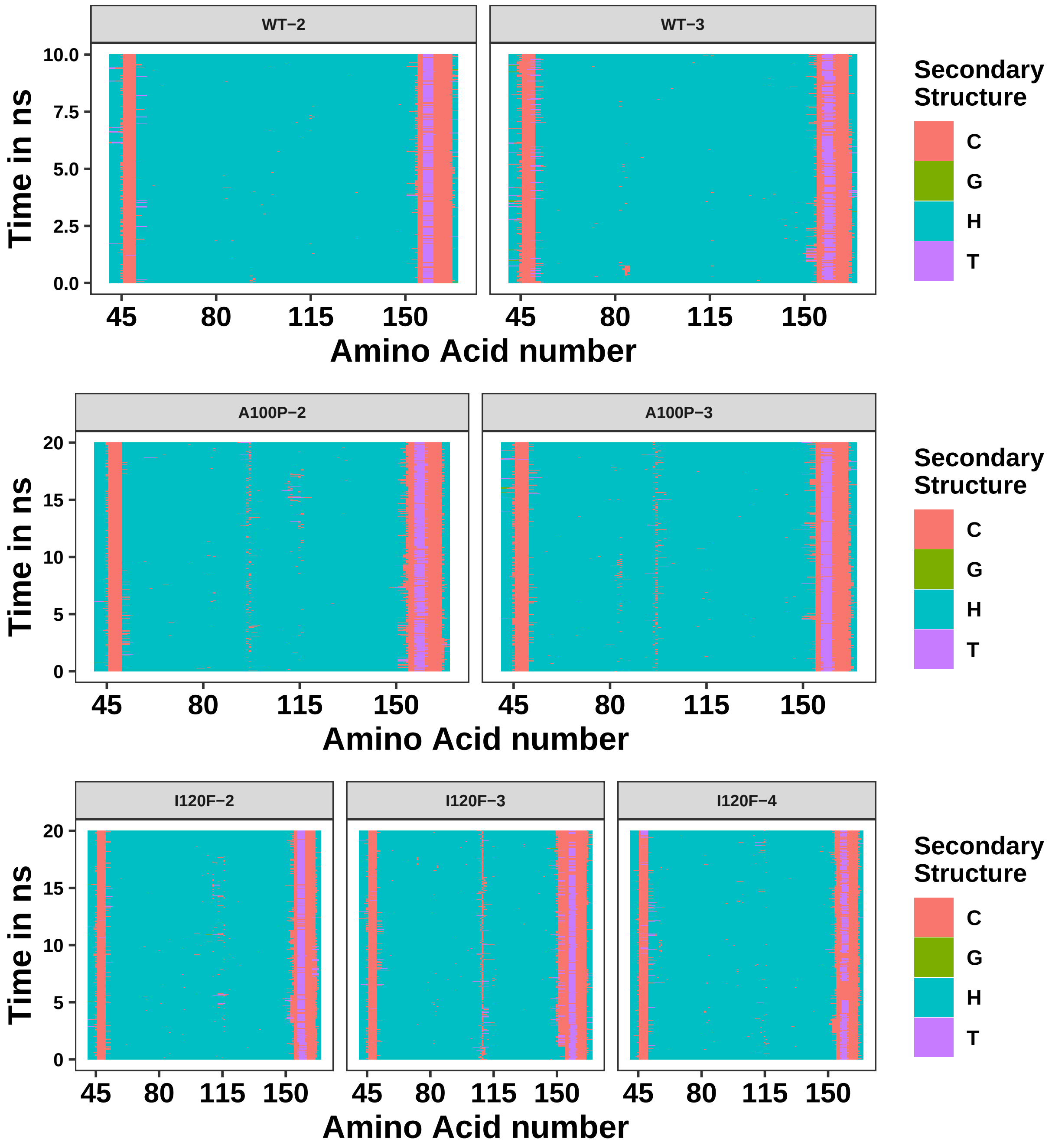

Figure 1: (A) Secondary structure over time plots from replicates of the WT, A100P and I120F simulations. Secondary structures were assigned by YASARA based on hydrogen bonding distances and psi angles at 25 picosecond intervals. 'H' stands for alpha helix, 'T' for turn, 'C' for coil, and 'G' for 3/10 helix.
