# Supplementary material for: Resilience of BST-2/Tetherin structure to single amino acid substitutions
Source: PeerJ. 2019 May 31;7:e7043. doi: 10.7717/peerj.7043 (PMC6546079; doi:10.7717/peerj.7043)
Supplement: Figure S2 — Differences in side chain position between the wild-type Isoleucine (purple) and the mutation Phenylalanine (yellow) for the 3 replicate simulations. Ile120Phe simulation (yellow) and the wild-type simulation (purple). Overlap is shown in grey. [file peerj-07-7043-s002.pdf]

Supplemental Figure 2

for Resilience of BST-2/Tetherin structure to single amino acid substitutions

by Ian R. Roy, Camden K. Sutton, and Christopher E. Berndsen

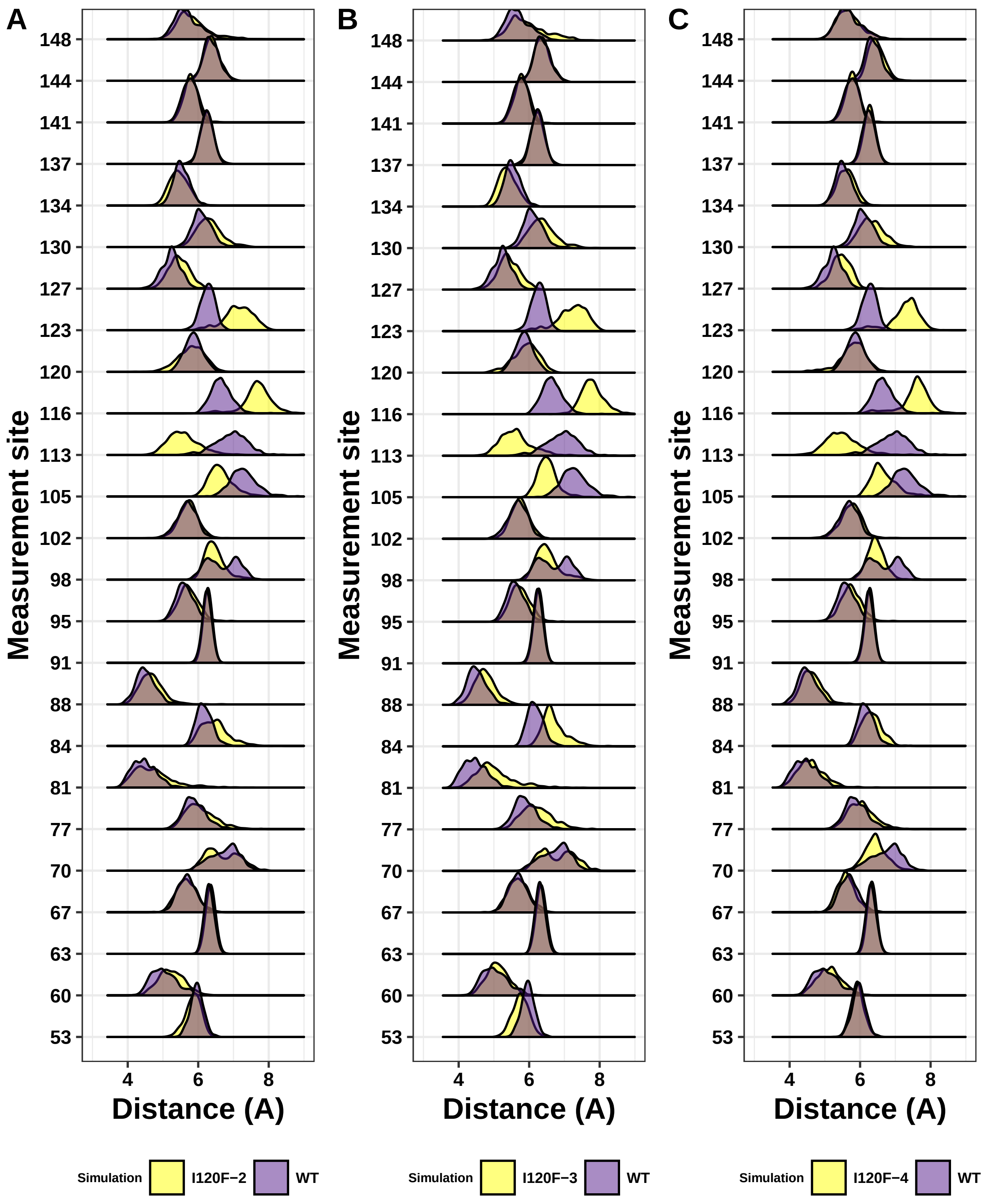

Figure 2: Differences in side chain position between the wild-type Isoleucine (purple) and the mutation Phenylalanine (yellow) for the 3 replicate simulations. Ile120Phe simulation (yellow) and the wild-type simulation (purple). Overlap is shown in grey
