# Supplementary material for: Resilience of BST-2/Tetherin structure to single amino acid substitutions
Source: PeerJ. 2019 May 31;7:e7043. doi: 10.7717/peerj.7043 (PMC6546079; doi:10.7717/peerj.7043)
Supplement: Figure S3 — (A) Distance between amino acids at the designated position plotted over the simulation time for the wild-type Tetherin. (B) Distance between amino acids at the designated position plotted over the simulation time for Tetherin with an I120F mutation. [file peerj-07-7043-s003.pdf]

Supplemental Figure 3

for Resilience of BST-2/Tetherin structure to single amino acid substitutions

by Ian R. Roy, Camden K. Sutton, and Christopher E. Berndsen

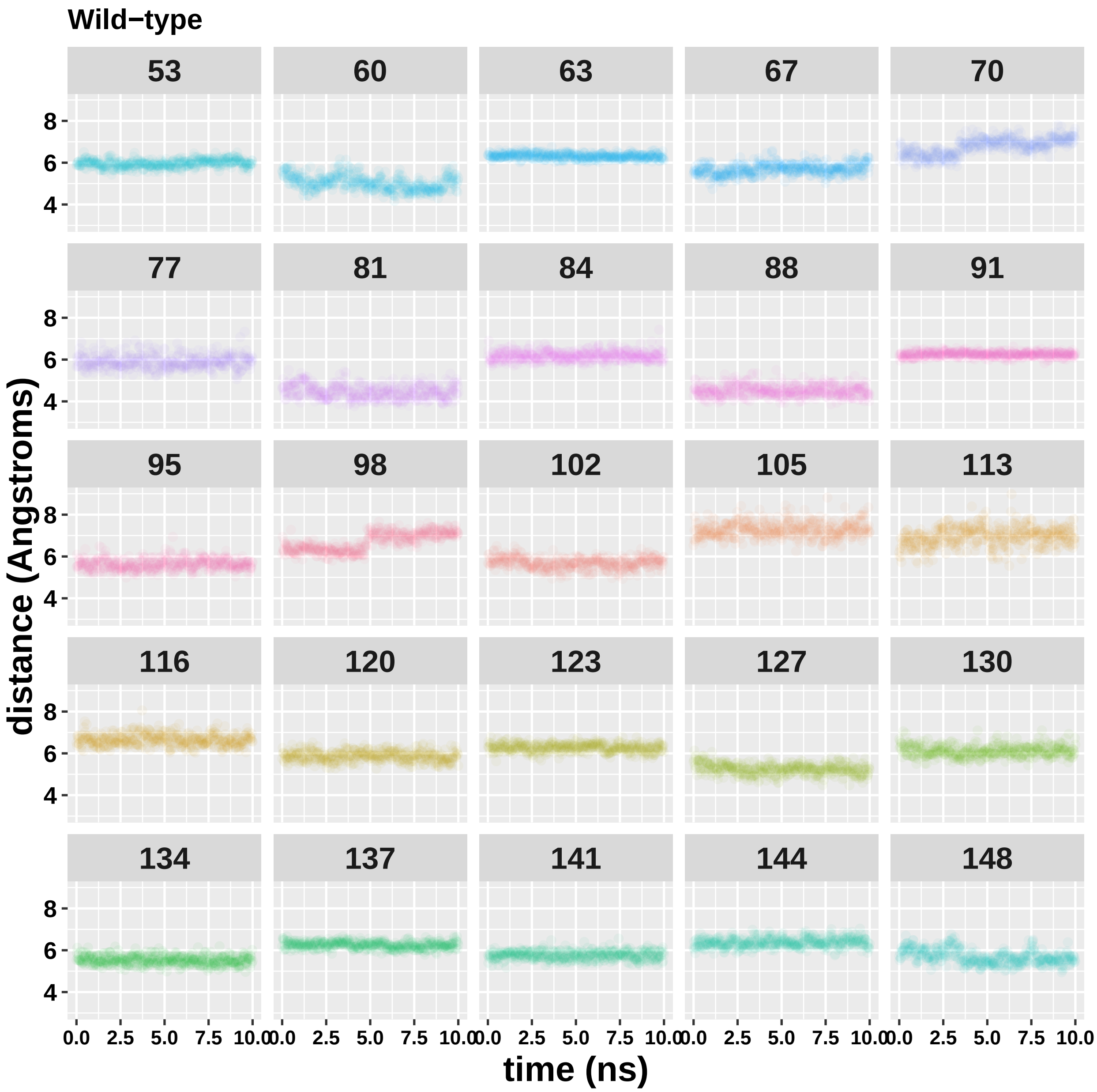

Figure 3A: Distance between amino acids at the designated position plotted over the simulation time.

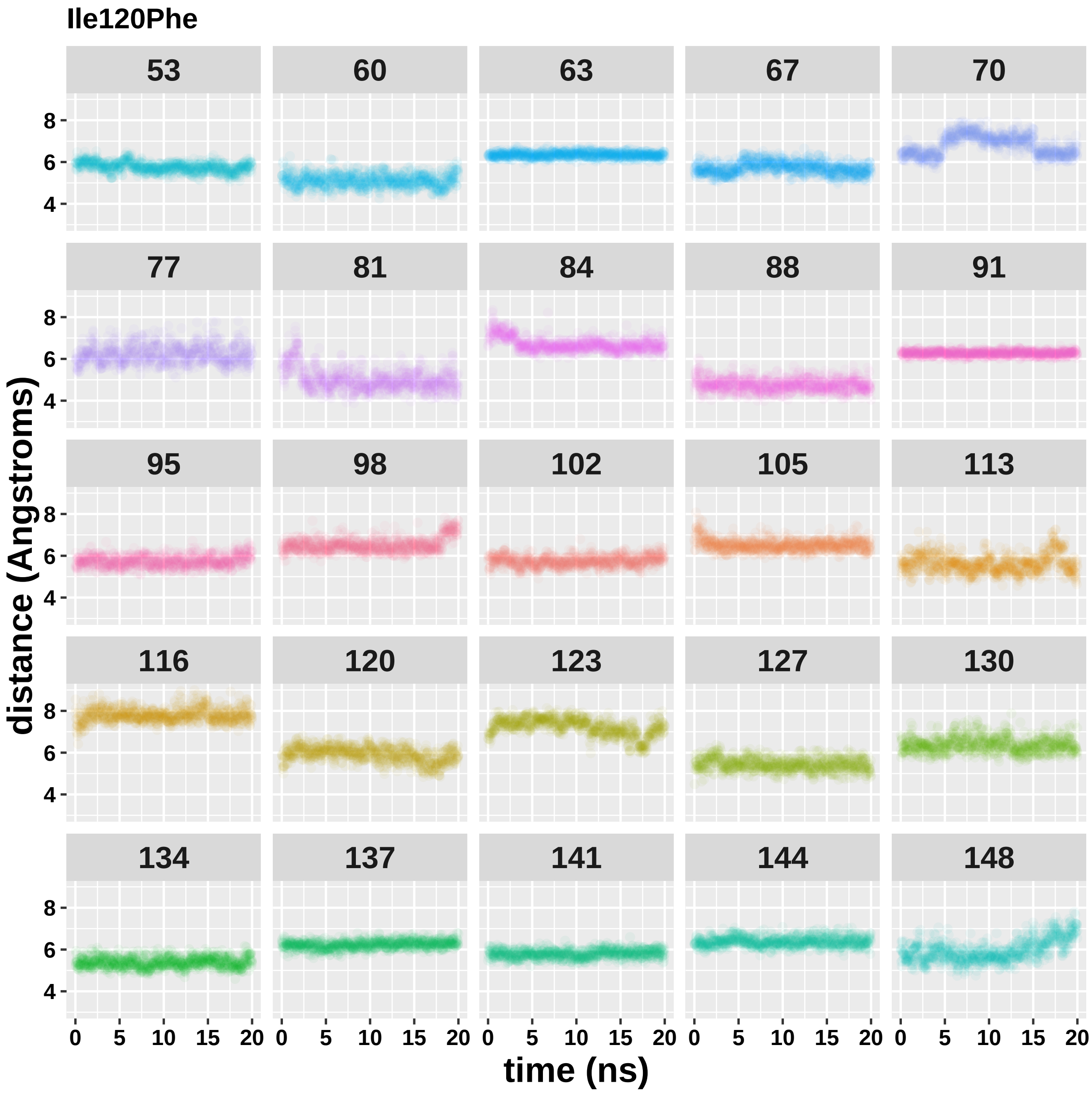

Figure 3B: Distance between amino acids at the designated position plotted over the simulation time for Tetherin with an I120F mutation.
