# Supplementary material for: Resilience of BST-2/Tetherin structure to single amino acid substitutions
Source: PeerJ. 2019 May 31;7:e7043. doi: 10.7717/peerj.7043 (PMC6546079; doi:10.7717/peerj.7043)
Supplement: Table S1 — Grid of each SNP modeled along with information on effects on structure, function, and frequency in the population. ND means not determined and NA means not in a known functional region of Tetherin. [file peerj-07-7043-s004.pdf]

Supplemental Table 1

for Resilience of BST-2/Tetherin structure to single amino acid substitutions

by Ian R. Roy, Camden K. Sutton, and Christopher E. Berndsen

Table 1: SNP description table

Amino Acid Number	Wt Amino Acid	SNP Amino Acid	Functional Region	Local Structural Defect	Functional Defect	Minor Allele Frequency	Frequency	Reference
49	Asn (N)	Ser (S)	NA	N	affects NF-kB activation, cell surface expression	< 0.01	2.27E-04	Sauter, et al., 2013
50	Ser (S)	Gly (G)	NA	N	ND	< 0.01	0.000259	NA
51	Glu (E)	Gly (G)	NA	N	ND	< 0.01	4.06E-06	NA
51	Glu (E)	Ser (S)	NA	N	ND	ND	ND	NA
52	Ala (A)	Ser (S)	NA	N	ND	< 0.01	4.06E-06	NA
52	Ala (A)	Val (V)	NA	N	ND	< 0.01	1.59E-05	NA
54	Arg (R)	Gln (Q)	NA	N	ND	ND	ND	NA
54	Arg (R)	Leu (L)	NA	N	ND	< 0.01	3.23E-05	NA
54	Arg (R)	Trp (W)	NA	N	ND	1.00E-02	1.91E-04	NA
55	Asp (D)	Asn (N)	NA	N	ND	< 0.01	4.06E-06	NA
56	Gly (G)	Ser (S)	NA	N	ND	< 0.01	2.03E-05	NA
58	Arg (R)	Gln (Q)	NA	N	ND	< 0.01	8.24E-06	NA
58	Arg (R)	Trp (W)	NA	N	ND	< 0.01	1.22E-05	NA
61	Met (M)	Ile (I)	NA	N	ND	< 0.01	8.12E-06	NA
61	Met (M)	Thr (T)	NA	N	ND	< 0.01	8.12E-06	NA
62	Glu (E)	Asp (D)	NA	N	ND	< 0.01	4.06E-06	NA
64	Arg (R)	Cys (C)	NA	N	ND	< 0.01	4.06E-06	NA
64	Arg (R)	His (H)	NA	N	ND	< 0.01	2.03E-05	NA
64	Arg (R)	Leu (L)	NA	N	ND	< 0.01	2.03E-05	NA
64	Arg (R)	Pro (P)	NA	N	ND	< 0.01	2.03E-05	NA
64	Arg (R)	Ser (S)	NA	N	ND	< 0.01	4.06E-06	NA
65	Asn (N)	Asp (D)	Glycosylation site	N	ND	< 0.01	4.06E-06	Andrew, et al., 2009; Waheed, et al., 2018
65	Asn (N)	Ser (S)	Glycosylation site	N	ND	< 0.01	1.50E-04	Andrew, et al., 2009; Waheed, et al., 2018
67	Thr (T)	Ile (I)	NA	N	ND	< 0.01	4.06E-06	NA
68	His (H)	Arg (R)	NA	N	ND	< 0.01	3.19E-05	NA
68	His (H)	Tyr (Y)	NA	N	ND	1.00E-02	4.06E-06	NA
69	Leu (L)	Phe (F)	NA	N	ND	< 0.01	8.13E-06	NA
71	Gln (Q)	His (H)	NA	N	ND	< 0.01	7.96E-06	NA
72	Gln (Q)	His (H)	NA	N	ND	ND	ND	NA
74	Leu (L)	Arg (R)	NA	N	ND	< 0.01	8.14E-06	NA
76	Glu (E)	Lys (K)	NA	N	ND	< 0.01	1.22E-05	NA
77	Ala (A)	Val (V)	NA	N	ND	< 0.01	4.07E-06	NA
82	Gln (Q)	Arg (R)	Proposed region of flexibility	N	ND	< 0.01	1.22E-05	Hinz, et al., 2010
83	Asp (D)	Asn (N)	Proposed region of flexibility	N	ND	< 0.01	4.08E-06	Hinz, et al., 2010
85	Glu (E)	Gly (G)	Proposed region of flexibility	N	ND	< 0.01	7.96E-06	Hinz, et al., 2010
86	Ala (A)	Ser (S)	Proposed region of flexibility	N	ND	< 0.01	1.23E-05	Hinz, et al., 2010
87	Gln (Q)	Lys (K)	Proposed region of flexibility	N	ND	< 0.01	0	Hinz, et al., 2010
88	Ala (A)	Thr (T)	Proposed region of flexibility	N	ND	< 0.01	4.10E-06	Hinz, et al., 2010
88	Ala (A)	Val (V)	Proposed region of flexibility	N	ND	< 0.01	8.24E-06	Hinz, et al., 2010
89	Ala (A)	Thr (T)	NA	N	ND	< 0.01	2.05E-05	NA
93	His (H)	Asp (D)	NA	N	ND	< 0.01	6.46E-05	NA
93	His (H)	Gln (Q)	NA	N	ND	< 0.01	4.13E-06	NA
100	Ala (A)	Pro (P)	NA	Y	ND	ND	ND	NA
101	Ser (S)	Phe (F)	NA	N	ND	< 0.01	8.13E-06	NA
103	Asp (D)	Asn (N)	NA	N	N	< 0.01	7.96E-06	Sauter, et al., 2013
107	Ala (A)	Asp (D)	Proposed region of flexibility	N	ND	< 0.01	4.06E-06	Ozcan and Berndsen, 2017
117	Glu (E)	Ala (A)	NA	N	N, alters Chugai Pharmaceuticals antibody binding	< 0.01	ND	Sauter, et al., 2013
120	Ile (I)	Phe (F)	NA	Y	ND	< 0.01	8.93E-06	NA
122	Thr (T)	Ala (A)	NA	N	ND	< 0.01	7.96E-06	NA
122	Thr (T)	Ile (I)	NA	N	ND	ND	ND	NA
125	His (H)	Arg (R)	NA	N	ND	< 0.01	1.33E-05	NA
127	Leu (L)	Val (V)	NA	N	ND	< 0.01	4.47E-06	NA
128	Gln (Q)	Arg (R)	NA	N	ND	< 0.01	3.24E-05	NA

Table 1: SNP description table *(continued)*

Amino Acid Number	Wt Amino Acid	SNP Amino Acid	Functional Region	Local Structural Defect	Functional Defect	Minor Allele Frequency	Frequency	Reference
129	Asp (D)	Asn (N)	NA	N	ND	< 0.01	4.51E-06	NA
129	Asp (D)	Glu (E)	Proposed region of flexibility	N	weak effect on viral restriction, alters eBioscience antibody binding	1.00E-02	3.63E-05	Sauter, et al., 2013; Ozcan and Berndsen, 2017
130	Ala (A)	Val (V)	Proposed region of flexibility	N	ND	< 0.01	4.58E-06	Ozcan and Berndsen, 2017
131	Ser (S)	Cys (C)	Proposed region of flexibility	N	ND	< 0.01	4.60E-06	Ozcan and Berndsen, 2017
134	Val (V)	Ala (A)	NA	N	ND	< 0.01	4.77E-06	NA
134	Val (V)	Met (M)	NA	N	ND	< 0.01	4.73E-06	NA
136	Arg (R)	Gln (Q)	NA	N	ND	< 0.01	4.89E-06	NA
137	Leu (L)	Met (M)	NA	N	ND	< 0.01	1.59E-05	NA
138	Arg (R)	Lys (K)	NA	N	ND	< 0.01	5.00E-06	NA
142	Gln (Q)	Arg (R)	NA	N	ND	< 0.01	4.11E-06	NA
143	Val (V)	Leu (L)	NA	N	ND	< 0.01	1.59E-05	NA
143	Val (V)	Phe (F)	NA	N	ND	ND	ND	NA
146	Val (V)	Leu (L)	NA	N	N	< 0.01	3.55E-04	Sauter, et al., 2013
147	Arg (R)	Gly (G)	NA	N	ND	ND	ND	NA
147	Arg (R)	Thr (T)	NA	N	ND	< 0.01	4.07E-06	NA
149	Ala (A)	Val (V)	NA	N	ND	< 0.01	8.15E-06	NA
150	Asp (D)	Gly (G)	NA	N	ND	< 0.01	4.07E-06	NA
151	Lys (K)	Asn (N)	NA	N	ND	< 0.01	4.07E-06	NA
153	Tyr (Y)	Cys (C)	NA	N	ND	< 0.01	8.14E-06	NA
154	Tyr (Y)	His (H)	NA	N	ND	1.00E-02	2.85E-05	NA
156	Ser (S)	Ile (I)	NA	N	ND	< 0.01	4.07E-06	NA
158	Gln (Q)	Glu (E)	NA	N	ND	< 0.01	3.23E-05	NA
159	Asp (D)	Ala (A)	NA	N	ND	< 0.01	7.96E-06	NA
161	Ser (S)	Cys (C)	NA	N	ND	< 0.01	4.07E-06	NA
163	Ala (A)	Thr (T)	NA	N	ND	< 0.01	1.22E-05	NA
