# Supplementary material for: Resilience of BST-2/Tetherin structure to single amino acid substitutions
Source: PeerJ. 2019 May 31;7:e7043. doi: 10.7717/peerj.7043 (PMC6546079; doi:10.7717/peerj.7043)
Supplement: Table S2 — Table showing the mutation site with information showing whether the mutation is in a known functional region, whether it causes defects in structure or function, and the associated reference. [file peerj-07-7043-s005.pdf]

Supplemental Table 2

for Resilience of BST-2/Tetherin structure to single amino acid substitutions

by Ian R. Roy, Camden K. Sutton, and Christopher E. Berndsen

Table 2: Designed mutant description table

Mutation Sites	Function Region	Local Structural Defects	Functional Defect	Reference
Asp55Ala Gly56Ala Leu57Ala Arg58Ala	NA	NA	Y	Hammonds, et al., 2012
Cys63Ala Asn64Ala Arg65Ala Val66Ala	Disulfide and Glycosylation sites	NA	Y	Hammonds, et al., 2012
Val74Ser Val84Ser Leu137Ser Leu144Ser	NA	NA	Y	Hammonds, et al., 2012
Arg54/58/64Asp	NA	NA	Y	Yang, et al., 2010
Glu76Arg Glu85Arg Asp83Arg	NA	NA	Y	Yang, et al., 2010
Glu76Arg Glu85Arg Asp83Arg Arg54/58/64Asp	NA	NA	Y	Yang, et al., 2010
Gln110Cys	Proposed region of flexibility	Y	Y	Welbourn, et al., 2016; Ozcan and Berndsen, 2017
Gly118Cys	NA	Y	Y	Welbourn, et al., 2016
